# Comparative Analysis of Systemic and Tumor Microenvironment Proteomes From Children With B-Cell Acute Lymphocytic Leukemia at Diagnosis and After Induction Treatment

**DOI:** 10.3389/fonc.2020.550213

**Published:** 2020-12-14

**Authors:** Geise Ellen Broto, Stephany Corrêa, Fausto Celso Trigo, Everton Cruz dos Santos, Fernanda Tomiotto-Pelissier, Wander Rogério Pavanelli, Guilherme Ferreira Silveira, Eliana Abdelhay, Carolina Panis

**Affiliations:** ^1^ Programa de Pós-graduação em Patologia Clínica e Laboratorial, Universidade Estadual de Londrina, Londrina, Brazil; ^2^ Laboratório de Biologia de Tumores, Universidade Estadual do Oeste do Paraná, UNIOESTE, Francisco Beltrão, Brazil; ^3^ Laboratório de Células-Tronco, Centro de Transplante de Medula Óssea (CEMO), Instituto Nacional de Câncer, Rio de Janeiro, Brazil; ^4^ Pediatrics Department, Hospital do Câncer de Londrina, Londrina, Brazil; ^5^ Programa de Pós-graduação em Patologia Experimental Universidade Estadual de Londrina, Londrina, Brazil; ^6^ Fundação Osvaldo Cruz, Instituto Carlos Chagas, Curitiba, Brazil; ^7^ Programa de Pós-Graduação em Ciências Aplicadas à Saúde, Universidade Estadual do Oeste do Paraná, UNIOESTE, Francisco Beltrão, Brazil

**Keywords:** interferon-gamma, interferon regulatory factor 3, transthyretin, tumor biology, proteomics, chemotherapy, B-acute lymphocytic leukemia

## Abstract

Among the childhood diseases, B-cell acute lymphocytic leukemia (B-ALL) is the most frequent type of cancer. Despite recent advances concerning disease treatment, cytotoxic chemotherapy remains the first line of treatment in several countries, and the modifications induced by such drugs in the organism are still poorly understood. In this context, the present study provided a comparative high-throughput proteomic analysis of the cumulative changes induced by chemotherapeutic drugs used in the induction phase of B-ALL treatment in both peripheral blood (PB) and bone marrow compartment (BM) samples. To reach this goal, PB and BM plasma samples were comparatively analyzed by using label-free proteomics at two endpoints: at diagnosis (D0) and the end of the cumulative induction phase treatment (D28). Proteomic data was available *via* ProteomeXchange with identifier PXD021584. The resulting differentially expressed proteins were explored by bioinformatics approaches aiming to identify the main gene ontology processes, pathways, and transcription factors altered by chemotherapy, as well as to understand B-ALL biology in each compartment at D0. At D0, PB was characterized as a pro-inflammatory environment, with the involvement of several downregulated coagulation proteins as KNG, plasmin, and plasminogen. D28 was characterized predominantly by immune response-related processes and the super expression of the transcription factor IRF3 and transthyretin. RUNX1 was pointed out as a common transcription factor found in both D0 and D28. We chose to validate the proteins transthyretin and interferon-gamma (IFN-γ) by commercial kits and expressed the results as PB/BM ratios. Transthyretin ratio was augmented after induction chemotherapy, while IFN-γ was reduced at the end of the treatment. Considering that most of these proteins were not yet described in B-ALL literature, these findings added to understanding disease biology at diagnosis and highlighted a possible role for transthyretin and IFN-γ as mechanisms related to disease resolution.

## Introduction

B-cell acute lymphocytic leukemia (B-ALL) is the most frequent neoplasia in childhood worldwide (1). Regardless of the great success in B-ALL treatment, this disease consists of an important cause of death in the child population. Considerable advances have been reached in understanding the biology of leukemia, and most are due to the scientific community efforts to bring new information based on high-throughput studies based on molecular approaches, that have driven recently B-ALL treatment to powerful targeted treatments (2).

The clonal rise of leukemia cells is mainly associated with cumulative mutations that directly affect the panel of proteins that are secreted in the tumor microenvironment (3). Some of these proteins are responsible for cellular events in the leukemic niche that control disease spread (4) and can result from systemic transcriptional changes related to disease prognosis (5). Drug therapy can also have a profound impact in B-ALL biology, further than killing its malignant clones. Changes in the local and systemic immunological profiles have been reported in B-ALL patients after completing conventional chemotherapy, and are related to their immune recovering (6). Remodeling induced by treatment in the genomic profile of blood cells from B-ALL has been reported as a determinant of prognosis and associated with disease outcomes as relapse and death (7).

Recently, proteomics-based approaches have been revealed as valuable tools to map changes in the protein profiling in both blood and bone marrow of B-ALL patients (8), and are helping to explore gaps related to disease relapse (9), chemoresistance (10), and biomarker discovery (11). Despite this, studies focusing on understanding the comparative analysis between the bone marrow tumor microenvironment and the peripheral blood are still missing. In this context, some overarching challenges as the hidden mechanisms behind immature lymphoid cells accumulation in the bone marrow, as well the mechanisms underlying the chemotherapy effects against B-ALL need to be improved, and therefore, the study of leukemia biology and the cumulative impact of the initial phase of chemotherapy become necessary.

For this purpose, this study performed the proteomic analysis of samples from children diagnosed with B-ALL by comparing bone marrow and peripheral blood profiles, before chemotherapy starting - at diagnosis, and the cumulative effects found after the induction treatment. To reach this goal, we used the nano-ultra performance liquid chromatography label-free proteomic strategy to obtain the differential proteomic profiles and further investigated the putative mechanistic evidence by using bioinformatics tools.

## Methods

### Patients Selection and Sample Collection

A total of 17 children diagnosed with B-ALL attended from September 2014 to January 2016 in the Londrina Cancer Hospital, Londrina-Paraná, Brazil, were enrolled. Those responsible for the children signed their consent terms. This study was approved by the Institutional Ethics Committee (approval number CAAE 24498213.0.0000.5231) and was designed and conducted following the ethical principles for medical research involving human subjects from the Declaration of Helsinki. The Reporting Recommendations for Tumor Marker Prognostic Studies (REMARK) criteria were followed regarding patient selection, assay performance, and data analysis throughout the study. All samples were routinely screened in the laboratory to determine the type of leukemia, and only those diagnosed as B-ALL were included in the study.

**Figure 1:**
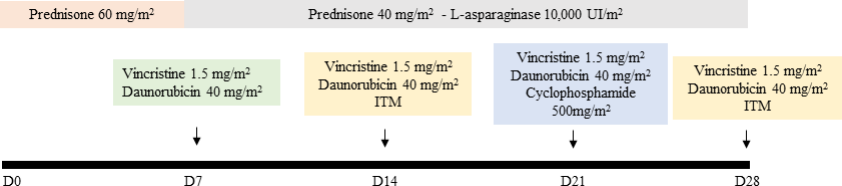
Induction phase chemotherapy schedule - Prednisone 60 mg/m^2^ orally from D1 to D7, then 40 mg/m^2^/day orally, divided into 2–3 doses for 3 weeks (D8-29), suspending regressively in 3 to 4 days. If necessary, Prednisolone can be administered intravenously, divided into 3 doses. Vincristine: 1.5 mg/m^2^/week intravenously maximum dose of 2 mg) administered on days 8, 15, 22, and 29. Daunorubicin: 40 mg/m^2^/week intravenously, administered on days 8, 15, and 22. L-Asparaginase: 10,000 IU/m^2^ intramuscular or intravenously (if thrombocytopenia <75,000/mm3) every 3 days, from day 8 of treatment, for a total of 9 doses. Cyclophosphamide: 500 mg/m^2^ intravenously on days 22 and 23 of induction for patients in the slow response subgroup. Intrathecal medication (ITM): triple therapy with Methotrexate, Ara-C and Dexamethasone will be administered at age-adjusted doses, on days 15 and 29 of induction (> 1 <3 years: 10mg/m^2^ and 20mg/m^2^, for Methotrexate and Ara-C respectively; > 3 <9 years: 12 mg/m^2^ and 24 mg/m^2^, respectively; > 9 years: 15 mg/m^2^ and 30 mg/m^2^, respectively. The dose of Dexamethasone is uniform (2 mg/m^2^. Max. 2 mg dose). Reference: Brazilian Group for Treatment of Childhood Leukemia scheme – GBTLI 2009 (12).

All patients presented as high risk at diagnosis, and were submitted to the same drug schedule, as determined by the Brazilian Group for Treatment of Childhood Leukemia scheme - GBTLI 2009, displayed in **Figure 1**. The induction protocol was based on a combined schedule based on prednisone, L-asparaginase, vincristine, and doxorubicin. Whole peripheral blood and bone marrow aspirate samples were collected in EDTA tubes from recently diagnosed patients, before starting any treatment (D0) and at the end of the chemotherapy induction phase (D28). Samples were centrifuged at 4,000 rpm for 5 min at 4^0^C to the obtention of plasma and kept frozen until analysis. Each sample was supplemented with a 1,000:1 (μl) protease inhibitor cocktail (GE Healthcare, USA).

### Label-Free Protein Quantitation *via* Mass-Spectrometry (MS)

Proteomic analysis was conducted with pooled plasma samples for each B-ALL group. Protein quantification was obtained *via* the Bradford assay, and the samples were processed using Amicon columns device of 3 kDa ultra-filtration (Millipore, USA) for concentration (39x) and exchanged buffer with 50 mM NH_4_HCO_3_. A total of 200 μg of protein were used for subsequent treatment with Rapigest 2 μg/μl (Waters), DTT 100 mM (Sigma), IAA 100 mM (Sigma), and tryptic digestion 0.25 μg/μl (High-grade Trypsin, Promega), carried out at 37°C, overnight and under light agitation (400 rpm). The proteomic approach applied in this study was the nano-Ultra Performance Liquid Chromatography (nano-UPLC) tandem nano-ESI-HDMSE method for qualitative and quantitative experiments. A nanoACQUITY UPLC system (Waters, UK) was employed, as previously reported by our group (13, 14). Briefly, a strong cation exchange column (180 μm × 23 mm, Waters, England) packed with Polysulfoethyl aspartamide (5 μm, PolyLC, USA) was used for the first dimension. Nine salt gradient fractions were used to elute the samples from the strong cation exchange column, followed by an RP gradient. After the peptides were captured, the trap column was placed online with a different RP analytical column (100 μm × 100 mm, 1.8 μm C18, nanoACQUITY UPLC HSS T3, Waters, UK), and an RP gradient of 5%–40% acetonitrile (containing 0.1% v/v formic acid) in 58 min was used as the second dimension, with a flow rate of 600 nL min−1. Analyses were performed using nano‐ESI in positive ion mode [nanoESI (+)] with a NanoLockSpray ionization source (Waters, UK). Multiplexed data-independent scanning with specificity and selectivity based on nonlinear “T-wave” ion mobility (HDMSE) experiments were performed with a Synapt HDMS mass spectrometer (Waters, UK), as previously described (15). Full scan orthogonal acceleration TOF (oa-TOF) MSE data were acquired from m/z 50 to 2,000.

### Database Searching, Quantification, and Statistical Analysis

Database searching and protein quantification were performed as previously reported (16–18). Using the PLGS Expression E tool algorithm, the identified proteins were organized into a statistically significant list corresponding to increased and decreased regulation ratios between the plasma from peripheral blood compared to plasma from bone marrow at D0 and the plasma from peripheral blood compared to plasma from bone marrow at D28. *In silico* analysis for biological processes, canonical pathways, network interactions, and transcription factors were performed using Metacore^™^ software (Clarivate Analytics, https://portal.genego.com/).

### Validation Study

Transthyretin and interferon-gamma (IFN-γ) were chosen as targets for the validation step. Transthyretin (also known as pre-albumin) was measured in PB and BM plasma by a commercial kit based on immune turbidimetry assay (Aptec Diagnostics, Belgium). Plasma samples were individually diluted 1:10 in a saline buffer and an aliquot of 50 µl was added to 900 µl of reaction buffer. This mixture was read at 340 nm (OD_1_). Then, 60 µl of goat anti-human pre-albumin antibody was added to this mixture and read again at 340 nm after a 5-min incubation (OD_2_). The difference between both OD was obtained, and transthyretin levels were calculated against a standard calibration curve. Interferon-gamma levels were determined in samples by using the Human Th1/Th2 cytokine kit (BD Biosciences, catalog number 550749) by flow cytometry. A plasma aliquot of 50 µl from each patient was mixed to 50 µl of capture beads provided by the kit and incubated for 3 h with 50 µl of PE detection reagent. After each tube was washed and centrifuged, the bead pellet recovered, plated, and read after incubation in a flow cytometer (BD Accuri™). The same procedure was performed to the calibration standard curve. Results were expressed individually and as PB/BM ratios, and compared by t-test in the software GraphPad Prism 6.0, considering as significant a p-value < 0.05.

## Results

**Table 1** shows the clinicopathological characteristics of patients. A total of 17 B-ALL children were enrolled in the study, the mean age at diagnosis was 6.8 years, 9 patients were male, most of them were Caucasian, and the mean body mass index was 16.8 kg/m^2^. The mean leukocyte counting in the PB at D0 was 18,273 cells/mm^3,^ranging from 200 to 74,300 white blood cells/mm^3^, and 1,609 cells/mm^3^ at D28 in PB, ranging from 200 to 9,700 white blood cells/mm^3^. The mean tumor cells counting in BM aspirate at D0 was 56%, ranging from 28%–90%, and 6.5% at D28, ranging from 0%–11%.

**Table 1:** Clinicopathological data of B-ALL patients.

	B-ALL patients
**Total number of patients**	n = 17
**Gender**	n = 8
Female	n = 9
Male	
**Ethnicity**	n = 15
Caucasian	n = 2
Afro-descendant	
**Mean age at diagnosis, years (min-max)**	6.9 (1.7–15)
**Mean body mass index, kg/m^2^ (min-max)**	16.8 ( 13.13–25)
**Mean leukocyte counting, peripheral blood cells/mm^3^ (min-max)**	18,273 (200–74,300)
D0	1,609 (200–9700)
D28	
**Mean blast counting in bone marrow aspirate, percentage (min-max)**	56 (28–90)
D0	6,5 (0–41)
D28	

**Figure 2:**
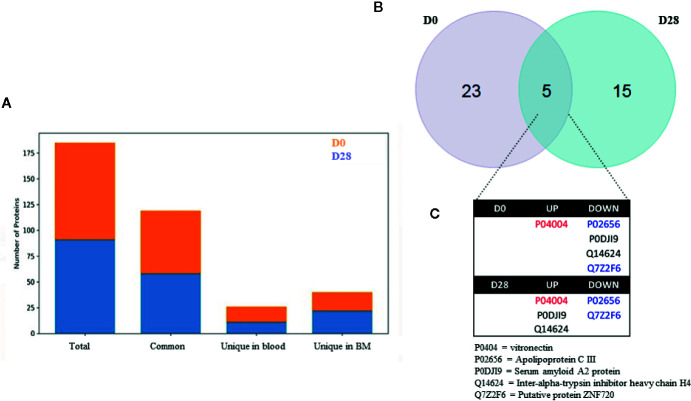
Proteomic design of the study **(A)** and the number of common and unique proteins identified in the high-throughput proteomic screening. In **(B)**, Venn’s diagram from the differentially expressed proteins in our study. Up and downregulated proteins differentially expressed in peripheral blood plasma samples from ALL-B patients at D0 – at diagnosis, before treatment start, and D28 – at the end of the treatment, comparing the blood versus the bone marrow. **(C)** Highlight from the 5 protein differentially expressed among D0 and D28. In red: upregulated proteins in both comparisons; In blue: downregulated proteins in both comparisons; In black: proteins in which quantification shifted according to comparison to bone marrow. * Chemotherapic protocol according to the Brazilian Group for Treatment of Childhood Leukemia scheme (2009).

**Figure 2** shows the results of the initial high-throughput screening of blood and bone marrow samples at D0 and D28. As shown in **Figure 2A**, 91 proteins were initially identified as differentially expressed, with 7 proteins expressed uniquely in the blood, 21 down expressed in blood, and 18 exclusively expressed in the bone marrow. At D28, 94 proteins were initially identified as differentially expressed among blood and bone marrow, being 8 overexpressed in blood, while 12 were down expressed in blood being exclusive from bone marrow. A false discovery rate (FDR) of a maximum of 4% was applied, and an FDR rate lower than 1% was detected for all analyses, at protein and peptide level, on average. Moreover, a minimum of 15 peptides, on average, was applied for protein identification.

The Venn diagram (**Figure 2B**) shows that 23 proteins were exclusively differentially expressed in the blood at D0, and 15 were exclusively differentially expressed in the blood at D28. Moreover, 5 proteins were common to both D0 and D28 (vitronectin, apolipoprotein C III, serum amyloid A2 protein, inter-alpha-trypsin inhibitor heavy chain H4 and putative protein ZNF720, **Figure 2C**). Vitronectin was upregulated in both comparisons whereas apolipoprotein C III and putative protein ZNF720 were downregulated in both comparisons. However, 2 proteins had their expression shifted among comparisons: serum amyloid A2 protein and inter-alpha-trypsin inhibitor heavy chain H4, which indicate their putative association in treatment response.

**Box 1** shows the final list containing differentially identified proteins after 2-fold change cut-off and statistical significance, and their status as up or downregulated in the blood at D0 and D28. The full list of identified proteins, together with raw data and details regarding methods were deposited in ProteomeXChange repository (Project accession: PXD021584, Project DOI: 10.6019/PXD021584).

Box 1 **Up and downregulated proteins differentially expressed in peripheral blood plasma samples from ALL-B patients at D0 – at diagnosis, before treatment start, and D28 – at the end of the treatment compared to bone marrow.**

**Table:** 

UPREGULATED IN BLOOD – D0
Uncharacterized protein C16orf90
Apolipoprotein C-I
Vitronectin
Inter-alpha-trypsin inhibitor heavy chain
Forkhead box protein E3
Dehydrogenase/reductase SDR family member 11
Protein RUFY3

**Table:** 

**DOWNREGULATED IN BLOOD – D0**
Apolipoprotein A-II
Immunoglobulin kappa variable 3D-15
Immunoglobulin kappa variable 3-15
Potassium channel subfamily K member 1
Putative PIN1-like protein
P antigen family member 1
Plasminogen
Kininogen-1
Hemoglobin subunit epsilon
Apolipoprotein C-III
Leucine-rich alpha-2-glycoprotein
Serum amyloid A-2 protein
Notch homolog 2 N-terminal-like protein C
Hemoglobin subunit gamma-1
Hemoglobin subunit gamma-2
Paired box protein Pax-2
Complement factor H-related protein 3
Inter-alpha-trypsin inhibitor heavy chain H4
Putative protein ZNF720
Notch homolog 2 N-terminal-like protein A
Protein eva-1 homolog A

**Table:** 

**UPREGULATED IN BLOOD – D28**
Transthyretin
Vitronectin
Serum amyloid A-2 protein
Inter-alpha-trypsin inhibitor heavy chain H4
Interferon regulatory factor 3
Reticulocalbin-3
Regulator of microtubule dynamics protein 1
Protein C10

**Table:** 

**DOWNREGULATED IN BLOOD – D28**
Homeobox protein VENTX
Prothrombin
Apolipoprotein C-III
Homeobox protein Hox-B1
Inorganic pyrophosphatase
ATPase family AAA domain-containing protein 3B
Docking protein 3
Putative protein ZNF720
Ribonuclease 8
Zinc finger and SCAN domain-containing protein 10
WW domain-binding protein 1-like
Hsp70-binding protein 1

**Figure 3:**
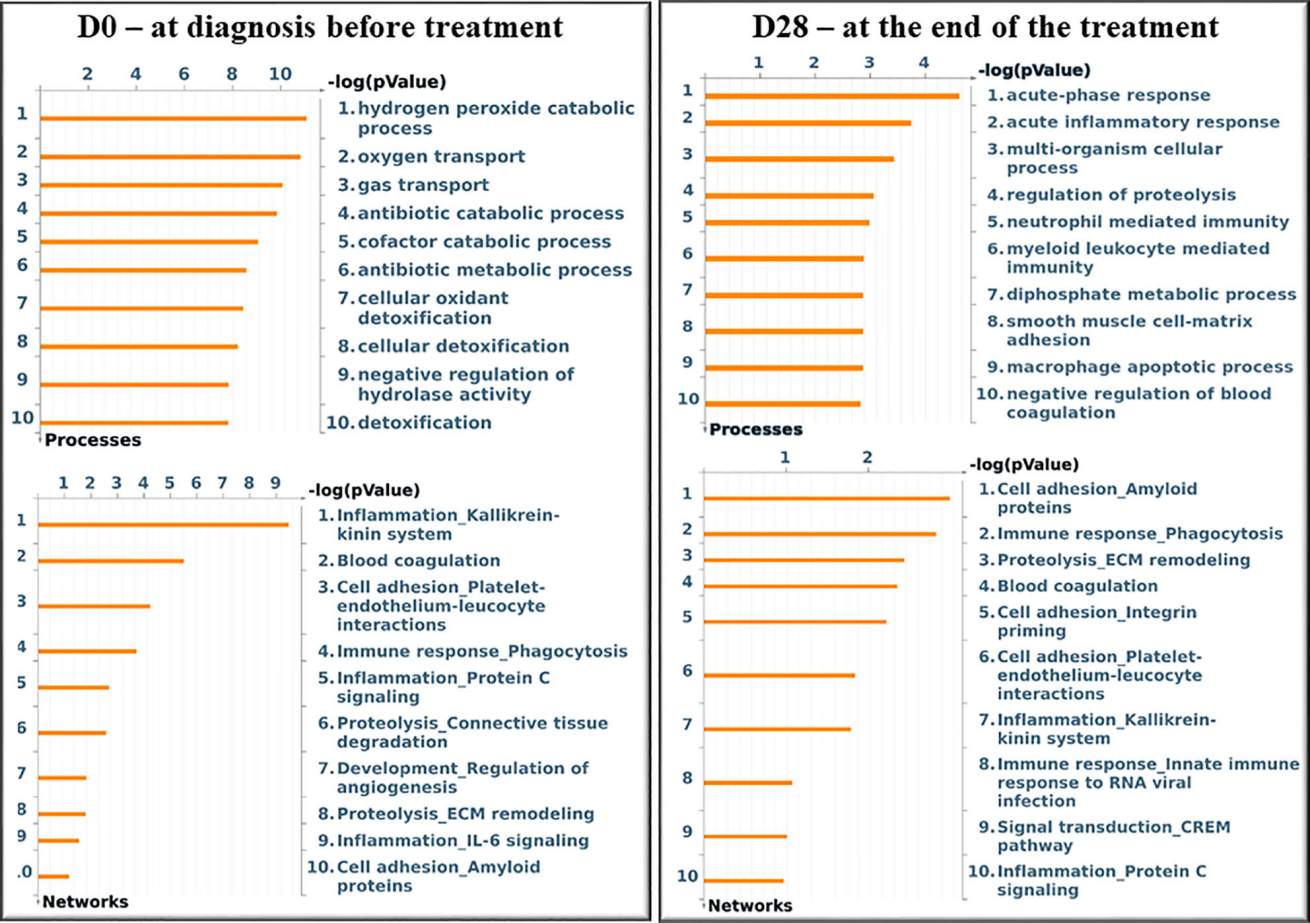
Significant Gene Ontology cellular processes and networks identified by Metacore analysis of the differentially identified proteins from each group. *In silico* analysis was performed using GeneGO Metacore™ software (GeneGO Inc., USA). Sorting is done for the ‘Statistically significant Processes.

**Figure 3** shows the significant gene ontology cellular processes and networks regarding the differentially expressed proteins in the blood at D0 and D28, as identified by using the Metacore software. At D0, the main processes identified were related to the cellular redox status and detoxification, and the networks were related to inflammation and blood coagulation signaling. At D28, the processes were related mainly to inflammation and immunity, and the networks represented mostly extracellular remodeling and innate immune response-related pathways.

**Figure 4:**
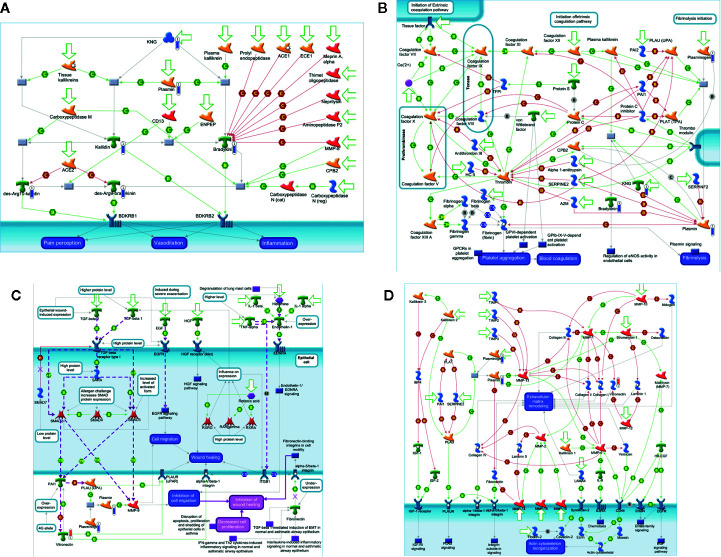
*In silico* analysis reveals the main biological networks differentially expressed in the blood of ALL-B compared to the bone marrow at diagnosis, before treatment start (D0). **(A)** Protein folding and maturation, **(B)** Blood coagulation, **(C)** Wound healing, and **(D)** extracellular matrix remodeling. Experimental data from all files is linked to and visualized on the maps as thermometer like figures. Upward thermometers have red color and indicate up-regulated signals and downward (blue) ones indicate downregulated expression levels of the genes.

The *in silico* analysis of the differentially expressed proteins at D0 in the blood of patients (**Figure 4**) revealed that some downregulated proteins, as KNG, plasmin, bradykinin, and kallidin, were enrolled in the protein folding and maturation process (**Figure 4A**), which affects inflammation. In **Figure 4B**, downregulated proteins from the coagulation system, plasminogen, KNG, and plasmin are shown. Plasminogen and plasmin are also highlighted in the map shown in **Figure 4C**, as participants of the wound-healing, proliferation, and migration processes. **Figure 4D** shows the involvement of the upregulated vitronectin in the extracellular matrix remodeling and actin cytoskeleton reorganization processes, altogether with the downregulated plasminogen and plasmin.

**Figure 5:**
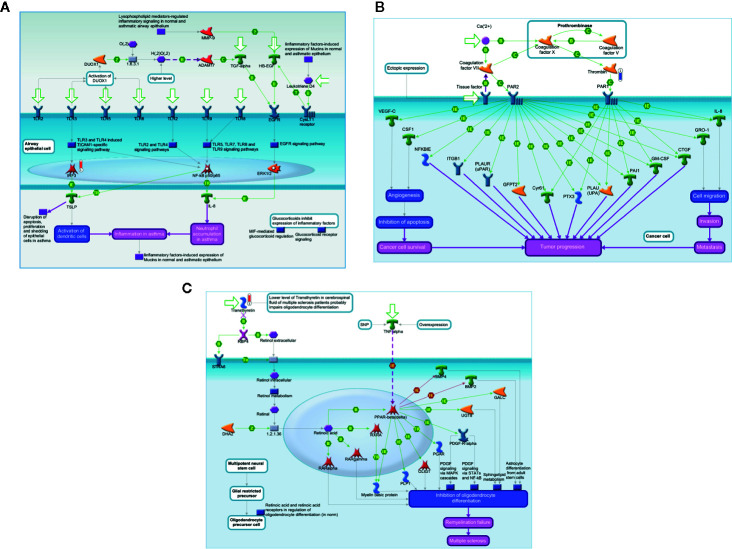
*In silico* analysis reveals the main biological networks differentially expressed in the blood of ALL-B compared to bone marrow in D28, at the end of treatment start. **(A)** TLR and EGFR-induced inflammatory signaling, **(B)** Expression targets of Tissue factor signaling in cancer, and **(C)** 7-Retinoic acid regulation of cell differentiation. Experimental data from all files are linked to and visualized on the maps as thermometer like figures. Upward thermometers have red color and indicate upregulated signals and downward (blue) ones indicate downregulated expression levels of the genes.

**Figure 5** shows the main biological networks for the differentially expressed proteins found in the blood at D28. **Figure 5A** shows the enrollment of the upregulated transcription factor named interferon regulatory factor 3 (IRF3) in triggering the TLR-inflammatory cascade. In 5B, thrombin downregulation and its putative role in cell migration and tumor progression pathways. **Figure 5C** demonstrates the upregulated protein transthyretin and its participation in the stem cell differentiation process.

**Figure 6:**
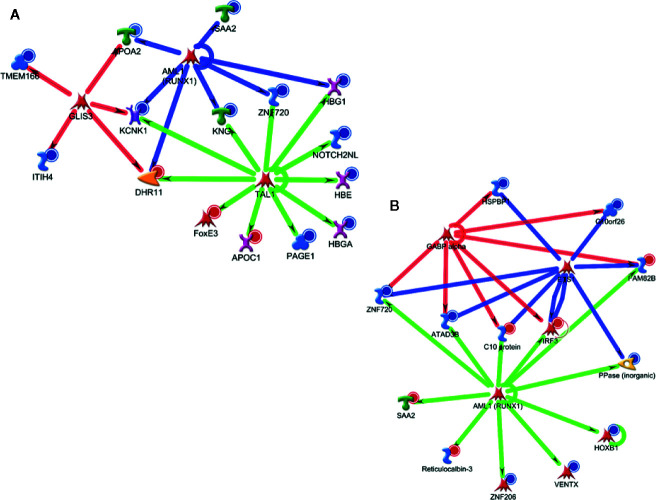
Major potential upstream regulators based on differentially expressed proteins at the blood in D0 **(A)** and D28 **(B)**.

Metacore analysis also provided the potential transcription factors (TFs) identified as upstream regulators in ALL-B identified proteins. As shown in **Box 2**, it was observed differences between the top 10 upstream regulators pointed for D0 and D28 in the blood. Some TFs presented a difference in the score (e.g., FOXP3) and some TFs were only associated with D0 data (e.g., TAL1) or D28 data (e.g., GCR). We selected TAL1 from D0 analysis and AML1(RUNX1) from D28 analysis to visualize their regulation in the attempt to better understand the potential regulation of these TFs (**Figures 6A, B**). We observed a crosslink with other TFs, which is different among analysis (D0 x D28), together with an activation/inhibition regulation.

Box 2 **Top 10 upstream regulators from D0 and D28, after comparison between peripheral blood and bone marrow, identified by Metacore analysis.**

**Table:** 

DO	D28
GATA1	GATA1
TAL1	AML1 (RUNX1)
SOX17	SOX17
HNF4-alpha	HNF4-alpha
FOXP3	ETS1
AML1 (RUNX1)	GCR
SOX2	GABP-alpha
GATA2	SOX2
c-MYC	FOXP3
GLIS3	Oct-3/4

GATA1, GATA Binding Protein 1; TAL1, T-cell acute lymphocytic leukemia protein 1; SOX17, SRY-related HMG-box 17 protein; HNF4-alpha, Hepatocyte nuclear factor 4 alpha; FOXP3, forkhead box P3 or scurfin; AML1 (RUNX1), Runt-related transcription factor 1 (RUNX1) also known as acute myeloid leukemia 1 protein (AML1) or core-binding factor subunit alpha-2; SOX2, SRY-Box Transcription Factor 2; GATA2, GATA-binding factor 2; c-MYC, c-MYC proto-oncogene; Basic Helix-Loop-Helix transcription factor (bHLH); GLIS3, GLIS Family Zinc Finger 3; ETS1, v-ets erythroblastosis virus E26 oncogene homolog 1; GCR, glucocorticoid receptor; GABP-alpha, GA-binding protein alpha chain; Oct-3/4, Octamer binding transcription factor 3/4.

**Figure 7:**
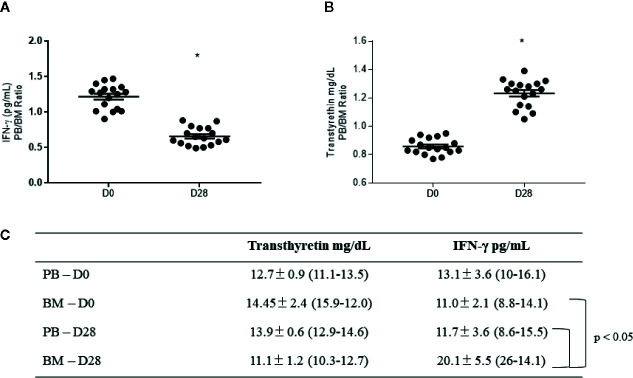
Validation of transthyretin and interferon-gamma (IFN-γ) levels in plasma samples from B-ALL patients. Peripheral blood (PB)/bone marrow (BM) ratio for **(A)** interferon-gamma and **(B)** transthyretin levels. In **(C)**, specific levels for each protein at diagnosis (D0) and the end of the treatment (D28), represented as mean ± standard error of the mean (min-max). * indicates statistical significance, p < 0.05.

For validation, we chose the proteins transthyretin and interferon-gamma (a protein originated from IRF3 activation), since both represent new information in B-ALL biology. Our data showed that the PB/BM ratio for IFN-γ was reduced at D28 (1.2 ± 0.1 at D0 and 0.65 ± 0.05 at D28, **Figure 7A**, p <0.05), while transthyretin was augmented at D28 (0.85 ± 0.05 at D0 and 1.25 ± 0.02 at D28, **Figure 7B**, p <0.05). Individual values for both transthyretin and IFN-γ measurements in PB and BM, at D0 and D28, are displayed in 7C (means ± standard errors of the means and min-max values). Finally, the individual comparison of data shown a significant augment of IFN-γ in BM at D28 (p <0.05).

**Figure 8:**
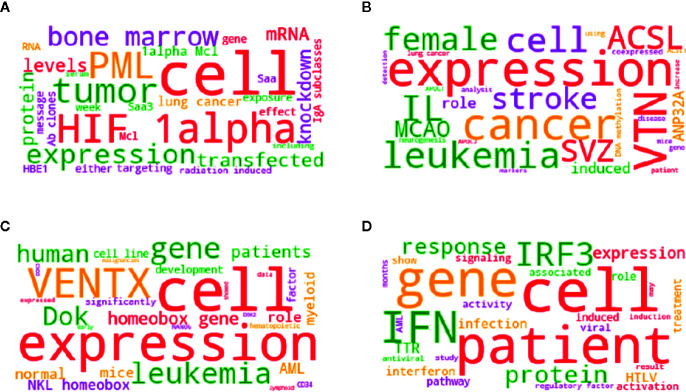
The group of proteins present in each time point and at different levels of expression, return frequencies of different terms in Pubmed search. Word cloud generated by the frequency of the terms present in the abstracts returned as a result of the search in the Pubmed. Four searches were carried out with the term leukemia and proteins in each analyzed condition [**(A)** - D0 down, **(B)** - D28 down, **(C)** - D0 up, and **(D)** - D28 up], returning the 10 most recent works. The abstracts of the works were concatenated and organized as a term corpus, analyzed by the Natural Language Toolkit package - NLTK 3.4 and WordCloud 1.6.0 for Python. The size of the word represents the frequency in the corpus.

To reinforce the relationship between the proteins discovered, we performed a word cloud study (**Figure 8**). The group of proteins present in each time point and at different levels of expression returned frequencies of different terms in a search in PubMed. Four searches were carried out with the term leukemia and proteins in each analyzed condition (A - D0 down, B - D28 down, C - D0 up, and D - D28 up), returning the 10 most recent works. The abstracts of the works were concatenated and organized as a term corpus (analyzed by the Natural Language Toolkit package - NLTK 3.4 and Word Cloud 1.6.0 for Python). The size of the word represents the frequency in the corpus and highlighted the interferon axis at the D28.

## Discussion

Proteomic-based strategies are powerful tools to identify new information in tumor biology studies, by using designs that are of relevance to clinical practice (19, 20). In the present study, the comparative analysis of the proteomic profile between the systemic (blood) and tumor (bone marrow) microenvironments provided a picture regarding the main proteins and processes that are present at diagnosis(D0) and triggered by the induction chemotherapy (D28) in the blood from ALL-B patients. The comparison performed between D0 and D28 allowed to describe the cumulative effect of cytotoxic treatment, understand the main processes present in ALL-B at diagnosis, and to know the main proteins differentially expressed that are relevant in each compartment before and after chemotherapy.

At diagnosis, before any treatment starting (D0), the blood was characterized as a pro-inflammatory environment, where some redox processes might be occurring. Further, it was identified that proteins that are classically viewed as coagulation players can also participate in different signaling pathways and processes in B-ALL. The involvement of coagulation proteins as KNG, plasmin, and plasminogen, that were found in our study as downregulated in the blood at diagnosis (D0), was suggested by *in silico* analysis as far beyond than clotting. The participation of such proteins in pivotal processes as wound healing and extracellular matrix remodeling has not yet been discussed in B-ALL biology.

The four main processes highlighted by the *in silico* analysis at D0 are cross-linked and display the systemic movement for tissue remodeling headed by coagulation proteins in the context of B-ALL. Protein folding processes refer to the tissue remodeling-driven events triggered by the exposure of tissue factors, in which coagulation cascade plays a crucial role. The extracellular matrix (ECM) is a broadly dynamic tissue structure that changes to allow processes as cell proliferation, migration, and differentiation (21). Therefore, at B-ALL diagnosis, it seems to exist a battle between pro and anti-ECM remodeling, headed by different proteins.

The downregulation of the coagulation proteins identified here could represent a tentative protective mechanism since the inhibition of wound healing processes could result in reduced cell proliferation and impair migration, both necessary in leukemia biology. It is known that dysregulation of ECM composition can critically support cancer progression and the malignant behavior of the cells by changing the interplay between the tumor microenvironment and the cells who reside there (22). Bradykinin, for example, can have its availability affected negatively by metalloproteinases (23), while the inhibition of plasminogen activation attenuates the metastatic behavior of breast cancer cells (24). Therefore, the downregulation of these proteins could be positive for tumor establishment.

On the other hand, vitronectin seems to act here as a pro-ECM protein, since its up-regulation could affect positively the reorganization of the cytoskeleton by matrix metalloproteinases (25), favoring cell organization to spread. On cancer stem cells, it has been shown that vitronectin is the component present in human serum that drives stem cell differentiation through an integrin-dependent mechanism, responsible for tumor formation (26).

At the end of the induction phase (D28), which represents the cumulative effect of all drugs administered in patients for B-ALL shutdown, we found that the main processes and networks were related to the immune-mediated inflammatory response. We have previously reported that chemotherapy can regulate negatively the systemic acute-phase proteins in breast cancer patients, as coagulation proteins, by using the same analytical strategy (27). The downregulation of thrombin found in our patients should be important for their remission since this protein is connected with angiogenesis, tumor growth, and metastasis (28).

Another important chemotherapy-induced event reported here refers to the super expression of the transcription factor IRF3 in the blood of B-ALL-patients, induced by chemotherapy. From the best of our knowledge, this is the first report that describes the presence of IRF3 in the blood of B-ALL patients. Despite the role of IRF3 in B-ALL is little known, it seems to be linked to B-cell differentiation in the presence of the TEL-AML1 fusion protein, the most common genetic rearrangement reported in B-ALL patients (29). Considering this, IRF3 could represent a positive mechanism induced by chemotherapy in the present study, since after killing the malignant clones of B-ALL, the main systemic signaling changed to cell differentiation. To understand the impact of chemotherapy on IRF3 activation and B-ALL dynamics during induction, we measured the levels of IFN-γ, a type I interferon, that can both regulate (30) and be regulated by IRF3 (31). The axis IRF3/IFN-γ has been described as a pivotal immune mechanism in anti-tumor responses (31). Our data have shown that, at the end of the treatment, there was a considerable reduction in the PB/BM ratio of IFN-γ. In this context, the possibilities are i) the IFN-γ consumption by immune cells against B-ALL, since at D28 the tumor cells were cleared from BM by chemotherapy (as shown in **Table 1**, mean tumor cells counting in bone marrow aspirate under 7%) and ii) that IRF3 super expression at D28 was enough to induce the production of IFN-γ by cells from BM niche, more than those in the PB (as demonstrated in the box of **Figure 7**).

This reasoning takes our discussion to another super expressed protein found in the blood of our patients, the transthyretin. This protein has a role in normal cell differentiation by providing adequate levels of thyroxin, a hormone requested for B-cell maturation (32). Transthyretin has been reported as low at B-ALL diagnosis, but progressively augments in ALL patients during the induction phase of treatment (33), suggesting that this protein can be induced by chemotherapy. Investigation of transthyretin levels at D28 shown that its PB/BM ratio is significantly higher than at D0, corroborating this hypothesis. Considering this, it could be suggested that transthyretin super expression after induction treatment could represent a positive factor associated with B-ALL eradication. High transthyretin observed at D28 was concomitant with the elimination of the immature malignant clones in both PB and BM in all patients, suggesting that its augment could represent an additional marker for tumor cell elimination during the B-ALL induction phase treatment.

Therefore, D28 proteomic profiling called attention to proteins that are enrolled in the resolution of B-ALL by regulating cell proliferation and differentiation homeostasis.

The *in silico* analysis evidenced the possible TFs related to disease (D0) and to the induction treatment (D28). This because (1) most of the shared TFs among the different endpoints (D0 versus D28) do not have the same representativeness, suggesting that their regulation is altered with treatment. As shown from our data, AML1 (RUNX1) exhibited different targets and crosstalk to other TFs, despite being a TF present in both D0 and D28 analysis. This TF is mostly in AML due to the ETV6/RUNX1 translocation and its relevance has been recently discussed in B-ALL (34, 35). However, the RUNX1 role is still not investigated for non-translocated tumors. Secondly, the “exclusive” TFs from each data may account for the more specific molecular changes in B-ALL before and after treatment. TAL1 (TAL BHLH Transcription Factor 1, Erythroid Differentiation Factor) has been described classically as associated with ALL-T and enrolled with MAPK-Erk Pathway (36) and NF-kappaB Signaling (37). However, it has been reported as methylated in specific cohorts (patients aged >9 years and in patients showing relapse), suggesting its potential prognostic value (38). Our proteomic data together with bioinformatics analysis corroborates this hypothesis.

Proteomics has been used as a valuable tool to understand B-ALL biology and propose putative new markers of clinical relevance. A study from Cavalcante et al. conducted in B-ALL patients after induction chemotherapy using mass spectrometry reported proteins shared to those that we highlighted in the present study, including components from the coagulation pathway, pointed out as candidates to follow up favorable responses after induction therapy (39). Moreover, substantial advances are being reached with this approach regarding determinant clinical features of B-ALL, as the relation between surface proteins expression and disease risk stratification (40), highlighting proteomics as a complementary tool that can help to guide decision making.

In conclusion, our proteomic study added to understanding B-ALL biology at diagnosis and highlighted some important proteins and processes that may contribute to our understanding of the mechanisms concerning the impact of chemotherapy on disease resolution. Our findings highlight the relevance of IRF3-IFN γ axis induction as a possible mechanism enrolled in disease resolution, and point out transthyretin as an upregulated protein induced by the induction phase of chemotherapy.

## Data Availability Statement

The datasets presented in this study can be found in online repositories. The names of the repository/repositories and accession number(s) can be found below: The full list of identified proteins, together with raw data and details regarding methods were deposited in ProteomeXChange repository (Project accession: PXD021584, Project DOI: 10.6019/PXD021584).

## Ethics Statement

The studies involving human participants were reviewed and approved by State University of Londrina/National Concil of Ethics - Conep. Written informed consent to participate in this study was provided by the participants’ legal guardian/next of kin.

## Author Contributions

CP and EA–conceptualization, methodology and investigation, data curation, and writing. FT and GB–conceptualization, patient selection, investigation, and writing. SC and GS–formal analysis and writing. All authors contributed to the article and approved the submitted version.

## Funding

Araucária Foundation, Grant No 026/2016-PRPPG; Programa de Pesquisa para o SUS – PPSUS, Grant 01/2016; Conselho Nacional de Desenvolvimento Tecnoloógico – CNPq, Grant Universal 14/2014; Coordenação de Aperfeiçoamento Pessoal de Nível Superior – CAPES.

## Conflict of Interest

The authors declare that the research was conducted in the absence of any commercial or financial relationships that could be construed as a potential conflict of interest.
